# Predicting county-scale maize yields with publicly available data

**DOI:** 10.1038/s41598-020-71898-8

**Published:** 2020-09-11

**Authors:** Zehui Jiang, Chao Liu, Baskar Ganapathysubramanian, Dermot J. Hayes, Soumik Sarkar

**Affiliations:** 1 Department of Economics, Iowa State University, Ames, IA 50011 USA; 2 Department of Energy and Power Engineering, Tsinghua University, Beijing, 100084 China; 3 Department of Mechanical Engineering, Iowa State University, Ames, IA 50011 USA

**Keywords:** Plant sciences, Computer science

## Abstract

Maize (corn) is the dominant grain grown in the world. Total maize production in 2018 equaled 1.12 billion tons. Maize is used primarily as an animal feed in the production of eggs, dairy, pork and chicken. The US produces 32% of the world’s maize followed by China at 22% and Brazil at 9% (https://apps.fas.usda.gov/psdonline/app/index.html#/app/home). Accurate national-scale corn yield prediction critically impacts mercantile markets through providing essential information about expected production prior to harvest. Publicly available high-quality corn yield prediction can help address emergent information asymmetry problems and in doing so improve price efficiency in futures markets. We build a deep learning model to predict corn yields, specifically focusing on county-level prediction across 10 states of the Corn-Belt in the United States, and pre-harvest prediction with monthly updates from August. The results show promising predictive power relative to existing survey-based methods and set the foundation for a publicly available county yield prediction effort that complements existing public forecasts.

## Introduction

In the 2001 Nobel Prize winning paper “The Market for Lemons^2^”, George Akerlof develops a model of a second-hand car market where asymmetric information exists, sellers know the quality of their cars but the buyers do not. Buyers offer a price based on the expected quality of the car, this price includes the possibility that the car is of poor quality and is less than the price of a high quality car. As a result, sellers of the high-quality cars worth more than the average price will exit the market, driving the proportion of low value cars up, thus pushing offer prices further down. Eventually, only “lemons” are left in the market and the market collapses. Akerlof shows that the key to this collapse is information asymmetry (i.e., in this case sellers have more information than buyers do).

Recently, many public crop production forecasts are becoming private. In 2015 and 2016, Descartes Labs provided publicly available corn yield predictions. These were within one bushel of the final number^3^. That same year the Lab formed a partnership with Cargill, a large grain trader, and Descartes stopped publishing the information. The partnership became public in 2018^4^. In 2016 and 2017, TellusLabs provided excellent pubic predictions of US corn yield^5^ . TellusLabs stopped publishing the yield forecast after merging with IndigoAg a major grain trader. Similarly in 2011, the futures market in pork bellies stopped functioning because the volume of trade had fallen significantly. One company, Smithfield, was producing and selling 30% of the bellies and had excellent information on worldwide demand and supply. The other traders could not compete with Smithfield’s knowledge base and they stopped trading. In 2019, the market moved before the USDA released its information^6^ . This suggests that some private forecasts knew what the NASS yield estimate would be. This is a classic example of the information asymmetry problem.

One solution to information asymmetry is to provide public information to all participants in the market at the same time. The United States Department of Agriculture (USDA) has been predicting national corn yield and production every year since 1964, providing consistent, publicly available and unbiased information to all stakeholders. The main method they use to create this information is survey based. Additionally, they deploy enumerators who make field visits in important corn production areas. The results from this traditional survey method is subjective since it is the farmer’s estimation of his/her yield at the point the survey is taken. USDA has tried new sources of data collection including satellite imagery from MODIS (moderate resolution imaging spectroradiometer) from NASA. However, as of fall 2018, USDA continues to rely primarily on the survey-based data.

Several private companies, such as Lanworth^7^, Tellus Labs^8^ and Climate Corp^9^, are probably in a position to improve on the USDA survey. In contrast with the monthly state-level prediction from USDA, these companies set up plant growth models based on weather information and expert knowledge, monitor satellite imagery and weather patterns, and incorporate as many independent lines of evidence as possible into their estimates to produce daily yield estimates of both individual farms as well as aggregated estimates at the county scale. Corn futures traders in the Chicago Mercantile Exchange who have preferential access to this information may be in a position to make profitable trades to the detriment of traders who do not have access. Corn futures prices have a strong negative correlation with expected corn yield. If a company or individual can predict yield more precisely, they will have better information about futures prices and can speculate better in the futures market, therein causing an information asymmetry problem.

Our motivation is to provide the public with a high-quality corn yield prediction that can substitute for private information from companies, thus eliminating information asymmetry in the corn futures market in the long run. We attempt to do so by improving the accuracy and quality of USDA predictions. Two limitations of USDA predictions are that they only offer state-level, but not county level predictions, and that USDA only publishes four monthly prediction reports annually instead of a daily early prediction during the corn-growing season. In this paper, we utilize advanced machine learning techniques to provide monthly Corn-Belt corn yield predictions at the county level. This method can also be used to provide a daily update on expected yield.

### Background knowledge

Corn is mainly grown in the midwestern part of the United State, in an area called the Corn-Belt. (Corn belt definition, https://digitalcommons.unl.edu/cgi/viewcontent.cgi?article=2848&context=usdaarsfacpub.) The region is characterized by level land, deep fertile soils, and high organic soil concentration^10^. The Corn-Belt includes Iowa, Illinois, Indiana, southern Michigan, western Ohio, eastern Nebraska, eastern Kansas, southern Minnesota and parts of Missouri. Corn is typically planted in April and harvested in October. The USDA reports the nationwide county level corn yield in late February of the following year. The USDA provides a monthly estimate of expected yield beginning in August. Farmers use the corn futures contract as a way to reduce risk. They accomplish this by selling a portion of their expected production at a set price before harvest commences. At the time of writing (7/30/2019) total volume of short (sold) futures on the Chicago corn futures market equaled 1,776,475 contracts^11^. Each contract is for 5,000 bushels. To put this in perspective this volume of sold corn represents approximately 60% of 2018 US corn production of 14.63 billion bushels^12^. The corn futures price depends heavily on the expected harvest volume, which in turn depends on the weather during the growing season.

### Related work

Beginning in the early 2000, groups started using traditional machine learning (ML) methods for yield prediction, and Basso and Liu review the recent progress on the yield forecast^13^. Basso et al.^14^ add spatial measurements—remote sensing in crop models. Remote sensing provides spatial inputs for the model and results show that a combination of crop model and remote sensing can identify management zones and causes for yield variability. Charles et al.^15^ use a spatial Bayesian regression model to predict maize yields in the Corn Belt. Though spatial smoothness among the regression coefficients will mitigate the effects of noisy data across regions and improve yield forecasting, their results indicate that corn yield prediction still remains a difficult problem. Gerlt et al.^16^ studied the relationship between farm-level yields and county-level yields by exploiting the fact that county-level yields are the aggregate of farm level yields to derive bounds that can be reduced to direct relationships between county- and farm-level yields under certain conditions.

Early research activities created ML models using discrete weather variables. These models, however, do not account for the long-term dependencies between (continuous) weather variables and final yield. A pressing question has been to develop and deploy methods that could provide insight into whether and how each point along the weather time series influences the final yield. In this regard, recent advances in data science and machine learning can help resolve this critical agricultural economics problem^17,18^. Here, we use Long Short-Term Memory (LSTM)^19^, a special form of Recurrent Neural Network (RNN)^20–22^ to accomplish this. The efficiency of LSTM networks in capturing long-term dependencies in multivariate time series with complex inner relations makes them a natural fit for this problem. Although LSTM is a promising and very popular deep learning approach, its use has remained particularly focused to applications in natural language processing. Our work is one of the first to apply LSTM in crop yield prediction, illustrating its potential for solving other prediction problems, and to improve the accuracy of publicly available corn yield prediction.

The selection of variables plays a prominent part in determining the quality of any predictive model. In this context, production agriculture contains a rich body of work that provides domain knowledge regarding important input variable in selection and preprocessing. For instance, the work^23^ (“The Seven Wonders of the Corn Yield World”), indicates that weather holds the first place among the seven “wonders” affecting corn yield. The key determinants of weather include rain, temperature, wind and humidity. Early work focused on fitting linear models to explore trends and patterns in crop yields associated with weather^24–26^. Recent work has shown that some weather variables (like temperature and water/drought) have nonlinear effects on corn yield^27,28^. There also exists another category of yield prediction using remote sensing^29^ which we do not consider for this study.

This sets the stage for ML models that can model both linear and non-linear effects without any hand crafting of parametrization. Some of the early work includes work by Monisha and Robert (2004) who used an artificial neural network (ANN) model with rainfall data to predict corn and soybean yield^30^. Newlands and Townley-Smith^31^ apply a Bayesian Network (BN) to crop yield prediction and used it to produce distributional forecasts of energy crop yield, and this was extended by Chawla et al.^32^ showed to predict county-level corn yield. More recently, Kim et al. (2016) use four machine learning approaches for corn yield estimation in Iowa—SVM (Support Vector Machine), RF (Random Forest), ERT (Extremely Randomized Trees), and DL (Deep Learning)—and utilize satellite images and climate data as explanatory variables^33^. With differences between their predictions and USDA statistics about 6–8%, they persuasively conclude that machine learning is a viable option for crop yield modeling. These prior efforts set the stage for a continuous variable based ML yield prediction model that can also provide in-season monthly updates to the predicted crop yield.

## Results

### Data collection

We collected publicly available historical data for ten states in the Corn-Belt: Iowa, Illinois, Indiana, Minnesota, Nebraska, Kansas, Michigan, Ohio, Missouri, and South Dakota. All data is from 1980 to 2016. The data range is restricted due to availability of weather data. The first 33 years are selected as the training data, while the most recent four years are used as testing data to explore the model’s predictive capability. As Iowa is the dominant corn planting state, we use it as our primary model testing state. The data collection process consists of two parts: collection of raw data and feature selection and preprocessing.

#### Yield data

**Figure 1:**
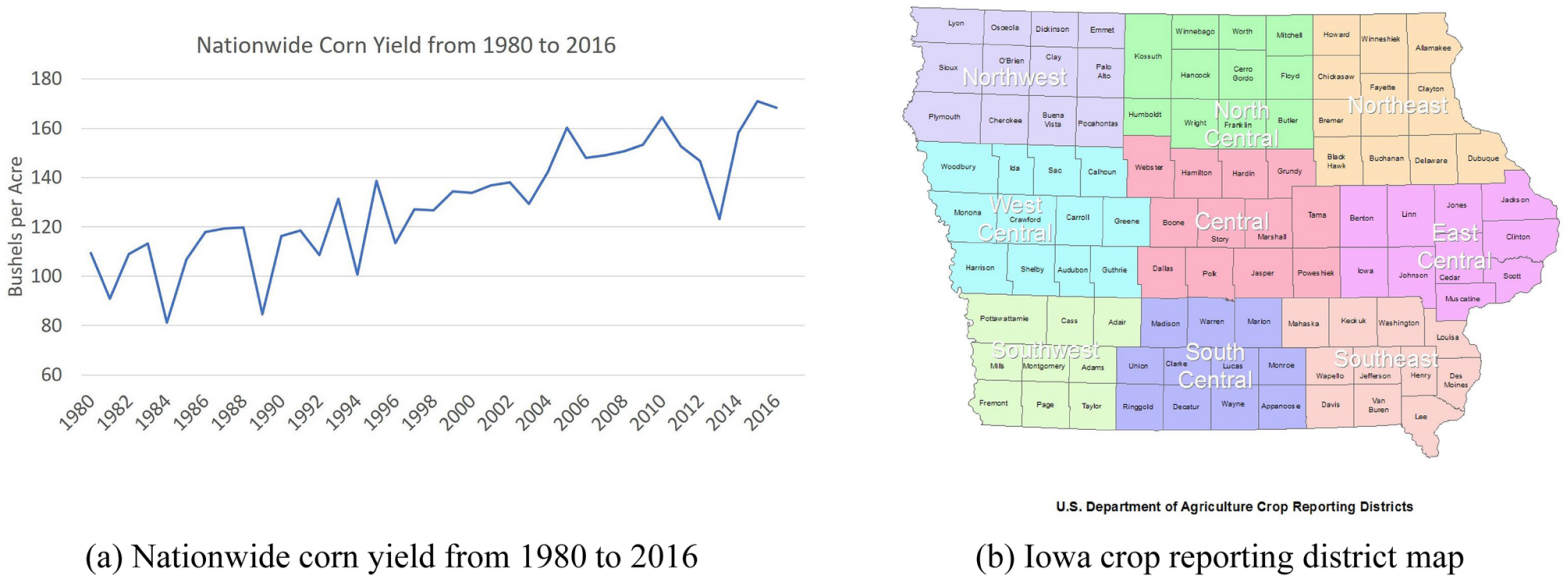
Background introduction: (**a**) Nationwide corn yield from 1980 to 2016, and (**b**) Iowa crop reporting district map (courtesy: https://www.icip.iastate.edu/sites/default/files/uploads/images/ region_maps/crop_districts.jpg).

Historical corn yield data is collected through Quick Stats from the National Statistics Service (NASS)^34^ for 37 years. Corn yield data is collected yearly at the county level. Taking Iowa as an example, this data consists of yearly information for each of the 99 counties in Iowa, resulting in 37 × 99 = 3,663 records of historical yield data available, with 3,267 of them acting as training samples. It should be emphasized that corn yields increase through time due to genetic gain (Fig. 1(a)). Therefore, we adjust the historical corn yield onto a uniform projected yield (similar to ‘inflation adjusted’ costs). We consider two methods of this adjustment: In the first approach we use a 1.5% annual yield increase, which is the standard number used by agricultural experts^35^. In the second approach, instead of a percentage increase, we use an absolute increase corresponding to genetic gain of 2.5 bu/ac per year from 1980 to 2000 and 4.67 bu/ac per year after 2000^36^. (We try both adjustments in our model; however, that raises a few more concerns. Which year should the yield be adjusted to? Will that influence the prediction results? If all yields are adjusted to the 2015 base year, will the 2015 prediction be better than other years? To answer these questions, we train our model with corn yield adjusted to both 2013 and the 2015 base year. The results show that there is no evidence for such concerns. Thus, we de-trend all the yield data into 2013 base).

#### Weather and soil data

Three types of input variables associated closely with corn yield are available. They are hourly weather data, soil quality data and soil moisture data. The hourly weather data was purchased from a weather data company—Weather Underground^37^. The data is a quality controlled and each data point is a 19 × 19 mile area spatially representative snapshot. We note that this is more accurate than the commonly used weather station data which measures weather at one central point. We use weather data from April to October to reflect the growing season in the Corn-Belt.

Weather variables impact yield in non-trivial ways. For instance, rainfall in the growing season may result in high yields, but extensive rainfall and flooding resulting in standing water can significantly reduce yield. Similarly, high wind speed can damage corn crops by uprooting plants, but in moderation can increase evapotranspiration. Temperature has the most complex impact on yield with the maximum, minimum and the mean temperature all influencing yields. Plant physiologists use a concept called Growing Degree Days (GDDs^)^^38^ to quantify this complex impact of temperature on plant development rates and yield. GDD are calculated as $GDD = \frac{{T_{\max } + T_{\min } }}{2} - T_{base}$ where $T_{\max }$ = min (86 °F, daily maximum temperature), and $T_{\min }$ = max (50 °F, daily minimum temperature). Here, $T_{base}$ is the minimal temperature (set to 50 °F for corn) over which photosynthesis is active triggering growth. Finally, we note that the cumulative GDD seen by the plant also influences the final yield.

Soil moisture has a critical impact on corn yield. The Palmer Drought Severity Index (PDSI)^39^ is a long-term cumulative measure of water availability in the soil. It is a standardized index that spans − 10 (dry) to + 10 (wet). 0 stands for a normal moisture condition, negative shows the soil is dry and positive means there is surplus water. PDSI uses temperature data and a physical water balance model to capture the basic effect of global warming on drought. PDSI is monthly data downloaded from the National Oceanic and Atmospheric Administration (NOAA) at the Crop Reporting District (CRD) level (Fig. 1b). We assign PDSI values to all counties within each district (i.e. counties in the same CRD has the same PDSI value).

**Figure 2:**
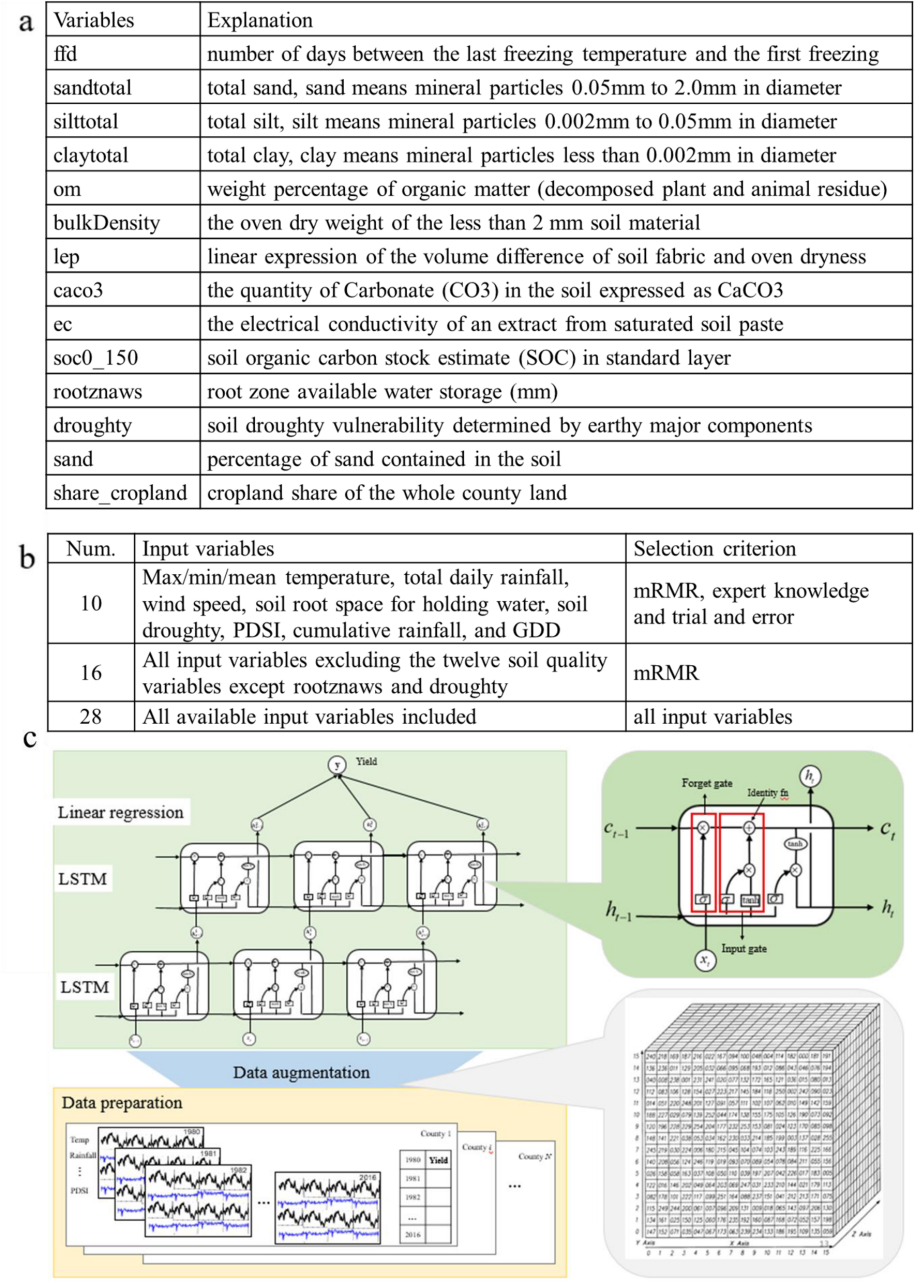
Data preparation and LSTM framework for yield prediction. The description of available soil variables is shown in (**a**), the three sets of selected variables are listed in (**b**) the LSTM framework is shown in (**c**) including data augmentation, the structure of LSTM unit, and the deep LSTM structure.

Finally, soil quality^40^ data is collected from the SSURGO database^41^ (database for storing gridded soil survey results) and aggregated to the county level using only areas classified as cropland according to the NLCD^42^ (National Land Cover Database). For continuous variables, we aggregated to the county level by taking the spatial average. Nathan Hendricks at Kansas State University completed this spatial averaging and kindly made it available to us. His procedure for averaging he data is described here^43^. The data covers the whole Corn-Belt region at county level. There are over one hundred variables in this dataset. Each variable is a constant number for each county, since soil quality typically does not change over time. We pick fourteen variables most related to corn yield (Fig. 2a) from this dataset. Among these, root zone for water storage and soil drought vulnerability are considered the most two significant soil variables.

### Variable selection and data preprocessing

The time series of input variables are expressed in a daily format. Each county for each year is one data point containing yield as the output variable and the corresponding input time series collected from April to October. Considered on a daily basis, the length of the input time series, $\{ x_{t} \}$, is t = 214. Choosing a finer granularity (i.e. hourly data) for the input was not promising due to two reasons, (a) with only 3,267 training samples, considering hourly time series (with a corresponding t = 5,136) let to too many parameters to viably estimate, and (b) daily weather data is able to track plant physiological response fairly well.

#### Variable selection

There are totally twenty-eight candidate input variables. In addition to the fourteen soil quality variables and PDSI, we also include max/min/mean temperature for each day, total daily rainfall, daily average wind speed, max rainfall during the day, accumulated rainfall and GDD accumulated up to that date. Since July is considered to be the most important month for corn growth^44^, rainfall and max temperature in July are also included. The ratio of acres planted for corn divided by the total acres planted may also help predict corn yield since farmers in corn intensive counties will specialize in corn management techniques and management. Two interaction terms (max temperature x soil droughty and max x PDSI) are also included. The idea behind this is that at high temperatures soil moisture will be more important than at low temperatures.

First, we trained the model using all these 28 variables. Our county level yield predictions turned out to be almost constant (at the county average). We subsequently used MRMR^45^ (minimum redundancy maximum relevance), a feature selection method introduced in Peng (2005). Using MRMR, we eliminated variables with low rank. Based on some additional model exploration and domain expertise, we arrived at the “best” ten input variables for corn yield prediction. These are max/min/mean temperature, total daily rainfall, wind speed, soil root space for holding water (rootznaws), soil droughty vulnerability (droughty), PDSI, accumulative rainfall and GDD.

#### Data augmentation

Even when using daily as opposed to hourly input series, 3,267 training samples are still not good enough for good training. A standard approach is to perform data augmentation. We perform data augmentation by considering pairs of nearby counties (in the same CRD) and take the average of their yield and input variables respectively, to create a new sample data. With this strategy, the total number of training data increases up to 70,026. (We emphasize that these combination samples are meaningful and reasonable since PDSI is also collected at the CRD level and all other data are also average numbers for county area (the most precise data point should be each farmland, which is not available).

Ten input variable sequences are stored in the format of 3D tensor cube for LSTM training. Figure 2 is an example of 3D tensor cube. X-axis indicates the number of the input variables, Y-axis is the length of the time series and Z-axis shows the number of samples. Hence the dimension of our 3D tensor is $10 \times 214 \times 70026$. (PDSI is monthly data, so it repeats the times of number of days in each month. While rootznaws and droughty repeat 214 times since they are constant).

### Yield prediction results

**Figure 3:**
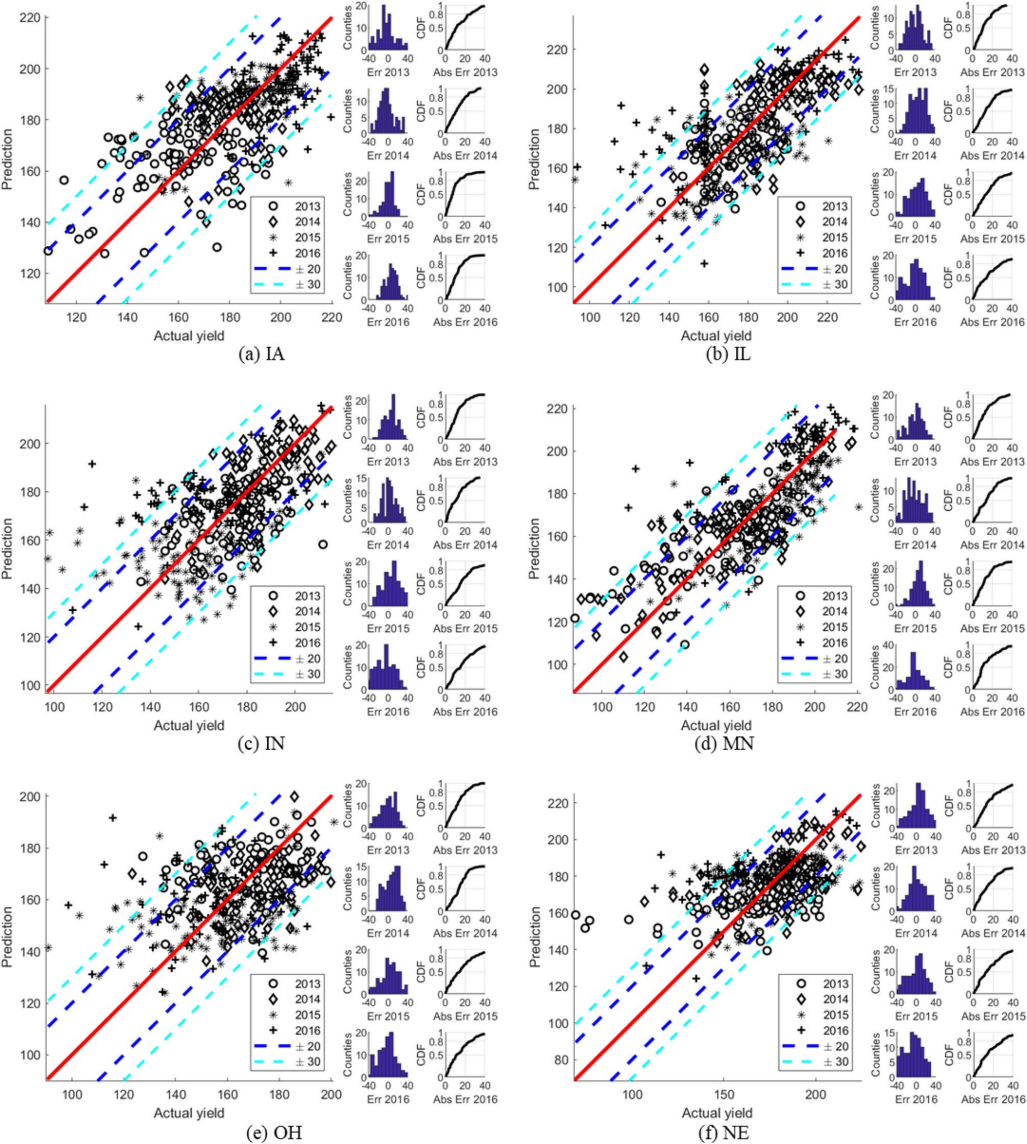
County-level Prediction Results in Iowa (**a**), Illinois (**b**), Indiana (**c**), Minneapolis (**d**), Ohio (**e**), and Nebraska (**f**). For each subplot, the left panel shows the prediction vs the actual yield and the error bars in 20 bu/ac and 30 bu/ac are marked in blue and cyan respectively. The right panel of each subplot show the errors of the predicted year (2013–2016).

We train separate models for each state with both percentage and constant adjustment. Our results indicate that percentage adjustment works much better for the whole Corn-Belt. Figure 3 shows our county-level prediction result for several states. Absolute errors between our prediction and the true value is calculated and represented as dots in the figures for each year. The results indicate that over 80% of the county-level yield prediction fall into the ± 20 bushels/acre region away from the true yield. We emphasize that this is a very significant result, especially considering that no farm management or genetic data is used.

We also aggregate the prediction to state level for each state to compare with the USDA prediction in November. In order to make the comparison clear, we only present the mean absolute error (MAE) and mean absolute percentage error (MAPE) of the prediction for 2013–2016. According to the results listed in Table 1a, all the MAPEs are less than 7% with most of them are less than 3%, indicating excellent predictive accuracy. The MAPE of Kansas, Missouri, and South Dakota was about 5–7%, these are the states that include less than 40 counties for testing. When we aggregate the results to the whole Corn Belt as shown in Table 1, the results are even better. The prediction error is less than 1 bushel/acre and also beats the USDA prediction.

**Table 1:** Corn yield prediction at State and Corn-Belt level. In (a), the state-level performance is evaluated with MAE and MAPE metrics, and the performance of NASS predictions are listed in the right panel for comparison. In (b), the Corn-Belt level performance of the proposed framework is evaluated yearly as well as the predictions by NASS.

State	Lasso_Nov		RF_Nov		AdaBoost_Nov		SVR_Nov		RNN_Nov		LSTM_Nov		NASS_Nov	
	MAE (bu/ac)	MAPE (%)	MAE (bu/ac)	MAPE (%)	MAE (bu/ac)	MAPE (%)	MAE (bu/ac)	MAPE (%)	MAE (bu/ac)	MAPE (%)	MAE (bu/ac)	MAPE (%)	MAE (bu/ac)	MAPE (%)
**(a)**														
Iowa	24.08	13.57	24.07	13.24	25.78	14.53	26.17	13.87	7.99	4.29	4.31	**2.29**	4.37	2.41
Illinois	19.41	10.03	13.04	6.73	11.16	5.64	8.55	4.61	11.59	6.05	2.85	1.56	3.50	1.92
Indiana	13.75	8.09	14.14	8.51	16.44	9.84	20.35	12.36	7.26	4.08	2.96	1.74	3.75	2.27
Kansas	12.45	9.81	18.88	14.60	17.74	14.03	8.36	6.58	19.13	13.58	10.14	6.95	4.25	3.02
Michigan	22.04	13.80	14.98	9.37	16.65	10.37	35.23	22.09	17.96	11.23	6.09	3.79	3.25	2.03
Minnesota	17.71	9.44	13.53	7.33	11.08	6.31	7.13	3.88	6.40	3.42	4.24	2.38	4.50	2.75
Missouri	20.29	11.76	23.25	14.74	16.03	9.96	28.51	17.18	28.30	17.36	9.92	5.88	3.25	2.06
Nebraska	9.60	5.31	4.62	2.56	8.42	4.65	8.24	4.60	8.44	4.68	3.41	1.95	2.50	1.39
Ohio	12.13	7.12	12.36	7.22	13.68	8.08	11.82	7.22	2.14	1.26	3.42	2.01	4.00	2.56
South Dakota	39.71	25.30	36.70	23.33	23.56	14.98	20.33	12.90	19.81	12.80	7.76	5.29	6.75	4.46

Year	Yield	Lasso_Nov	RF_Nov		AdaBoost_Nov		SVR_Nov		RNN_Nov		LSTM_Nov		NASS_Nov	
**(b)**														
2013	161.78	164.12	165.03		168.79		155.16		161.28		162.90		164.29	
2014	175.60	167.68	173.07		173.17		163.18		166.34		175.44		178.49	
2015	173.10	170.84	176.66		178.81		169.45		169.21		173.40		173.04	
2016	181.09	174.56	182.06		180.85		173.40		175.95		179.34		181.54	
MAE (bu/ac)		4.76	2.58		3.85		7.60		4.70		0.83		1.48	
MAPE (%)		2.72	1.51		2.29		4.38		2.67		0.48		0.87	

### Early prediction

**Figure 4:**
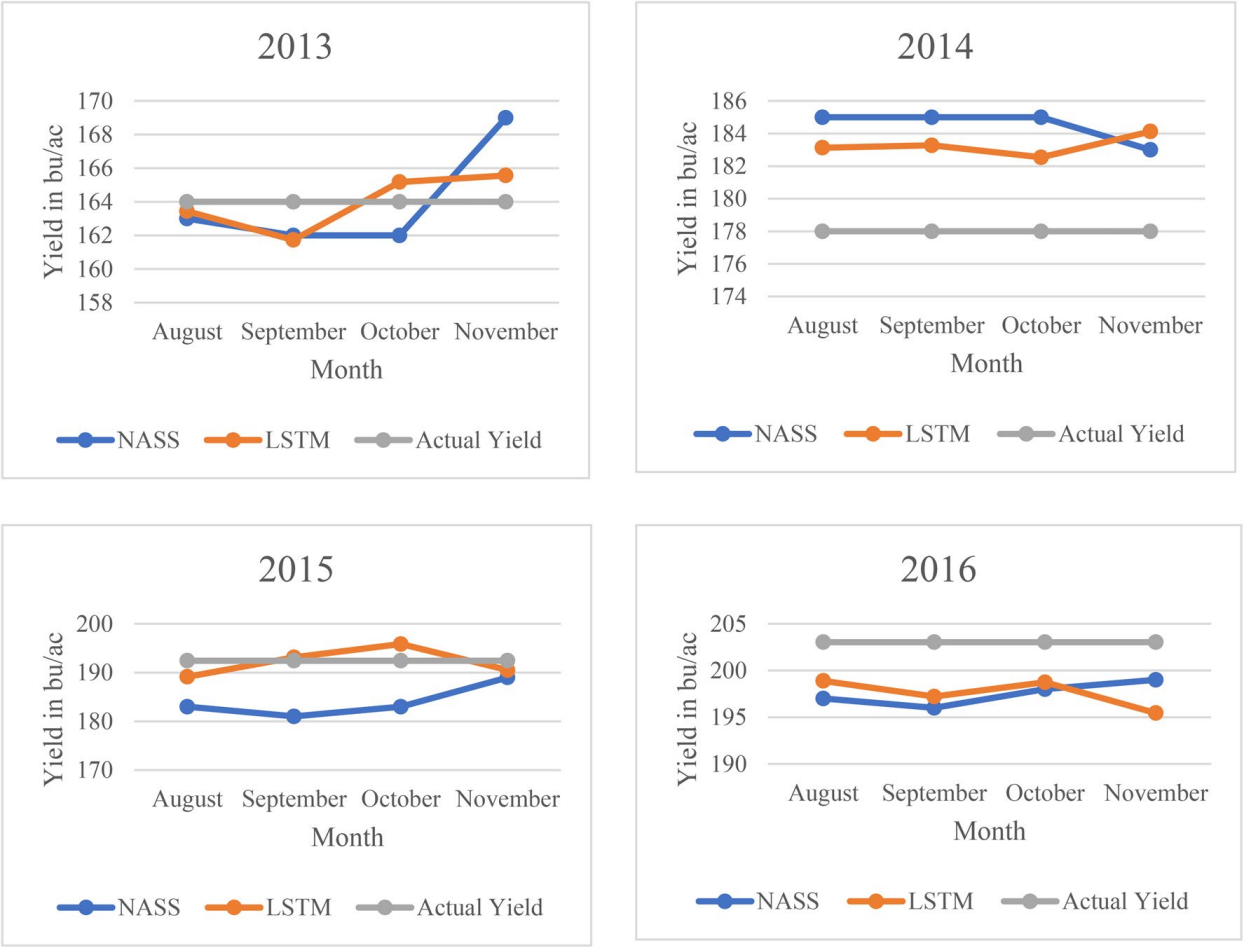
Early Yield Prediction Comparison between USDA and LSTM. The yield predictions from August to November by the proposed framework are compared with actual yields and the NASS prediction.

In order to make a comprehensive comparison between USDA and our model, we retrained our model for Iowa using three sets of partially available information, specifically with data available until August, September and October respectively (i.e. y = 122, 153 and 183 for the 3D tensor cube). These trained models make in-season predictions of final yield based on information available to them until that point. Figure 4 summarizes the prediction results of USDA and our LSTM model. It is clear that most of our early prediction results are better than the USDA prediction. We also observe that predictions in November are usually worse than in October. This is because we do not know the accurate harvest date for each year and data after the harvest date is included in the training data. Therefore, the resulting redundant and noisy information have been learned by the model and that influences the final prediction. This comparison indicates the power of our model in early prediction and the ability to beat USDA with limited data. (LSTM is also available for daily prediction. It would be too much work to train separate models for each day to make daily predictions. Therefore, we can use the best model for each state, input known explanatory variables data, and fill unknown data with expected values. Weather and soil humidity data after the date on which the daily prediction is made is the unknown data. The expected input values can be either past 10-year average or from the professional prediction in weather websites/channels if it is available).

### Performance comparison

For performance comparison with other ML techniques, Lasso, Random Forest, AdaBoost, Support Vector Regression (SVR), Recurrent Neural Network (RNN) approaches are also applied with the same training and testing data, and the results are listed in Table 1. Hyperparameter searching is implemented for every compared technique with a similar sized parameter search space as done for the LSTM model. The results show that the LSTM model outperforms for predicting yield in most of the states (Table 1a). At the corn-belt level (Table 1b), the LSTM model presents a much smaller MAE and MAPE, in comparison with the other listed methods. This verifies the efficacy of the proposed framework. A key aspect to note here is that while LSTM (as well as the RNN) model considers the temporal dynamics of the weather variables to forecast the yield at the end of the season, many other techniques, e.g., Lasso and Random Forest, do not explicitly capture that. We observe that these methods perform significantly worse compared to RNN or LSTM. Our LSTM model outperforms the RNN as well most likely due to the larger model capacity. Therefore, it is evident from our observation that explicit consideration of the temporal dynamics is critical for the yield forecasting problem.

## Discussion

This paper describes the prediction of county-level corn yields in the Corn Belt area using the deep learning method called LSTM. We only leverage historical weather and publicly available yield data to learn such models that are demonstrated to be a powerful option for crop yield prediction. Results show that our LSTM model can provide useful early prediction and accurate county-level corn yield prediction in the US Corn-Belt without private farm management data and the genetic information of seeds. As USDA NASS reports their yield prediction based on surveys, the NASS predictions in November already include a part of the harvesting data for some states such as Kansas and Missouri. In contrast, our model does not use such information, which is probably the reason for lower accuracy values for these states. Also, irrigation operations are widely used in Kansas, which would affect the yield. Our model does not use any farm management information such as irrigation, which also possibly influences the performance for this state. Finally, for Kansas, Missouri, and South Dakota, there are less than 40 counties that leads to smaller data sizes for these states compared to other states in the corn belt. Smaller data sizes cause difficulty in training complex deep learning models with large number of trainable parameters thus resulting in larger errors for these states.

**Figure 5:**
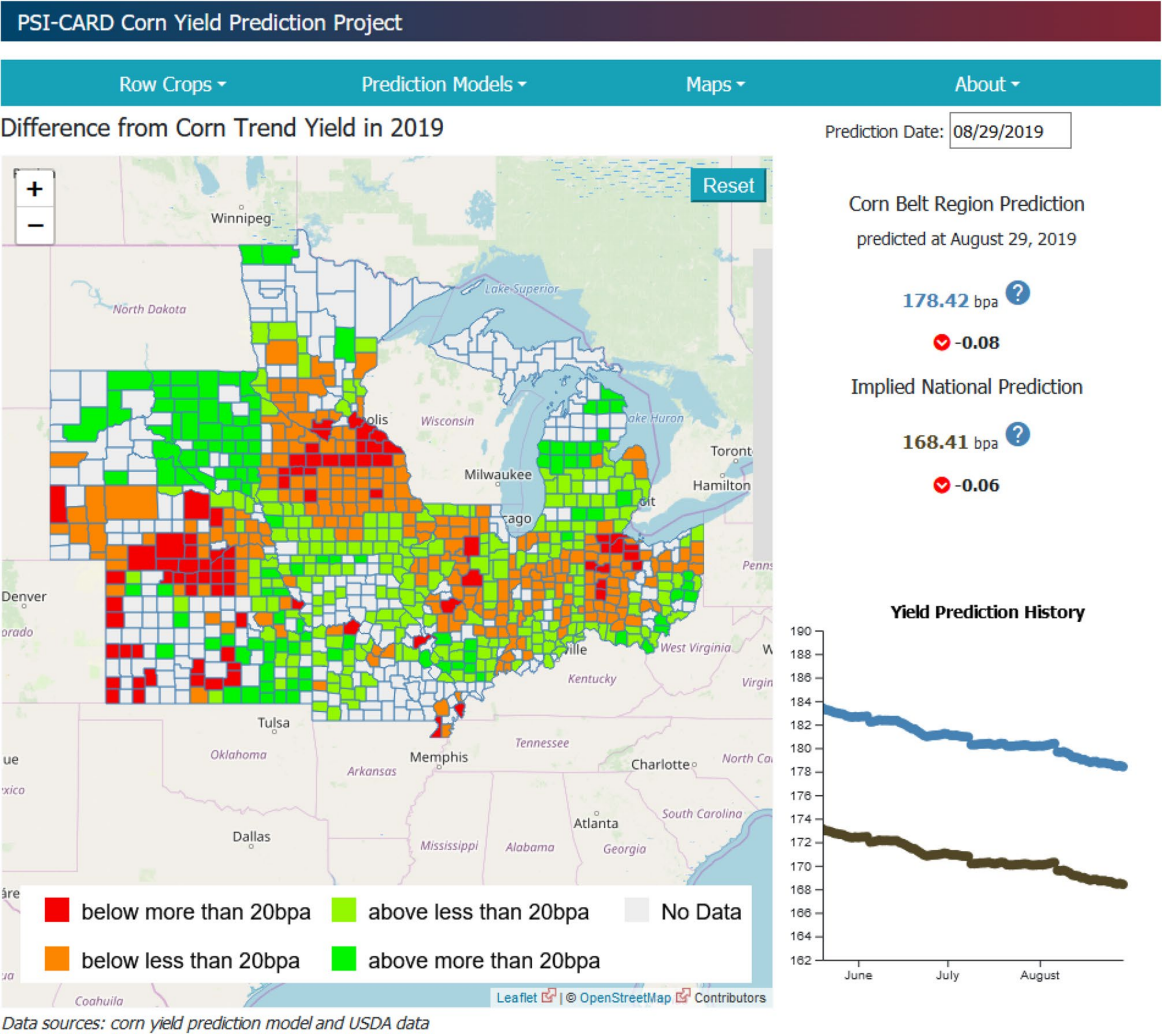
Screen shot from the public corn yield projection website established based on the proposed framework. The model proved to be extremely accurate, coming within one bushel of the true number in 2019 (courtesy: https://www.card.iastate.edu/tools/yield-prediction/, downloaded on 8/29/2019).

In summary, despite the lack of critical but non-public information for necessary for yield prediction such as farm management data, our proposed deep LSTM model shows excellent performance in predicting corn yield compared to other machine learning models and the NASS prediction for most cases. Therefore, our LSTM models seem to be a good supplement and improvement to the USDA prediction that will contribute to eliminating the information asymmetry problem that arises from the success of private companies in crop yield prediction. The authors established a public corn yield projection website and used hourly weather data to make available; county yield, Corn-Belt yield and national yield projections on a daily basis during the 2019 growing season. The model proved to be extremely accurate, coming within one bushel of the true number in 2019. A screen shot of the yield prediction from 2019 is provided in Fig. 5. The intent is to continue this effort using the website of Iowa State University, a major Land Grant University.

Several avenues for future work are possible. For instance, in using soil and weather information we assume that both crop land and weather are uniformly distributed throughout the county. This assumption can be relaxed by using spatial masks that enable weighted averaging of soil, cropland and weather to more accurately reflect weather and soil impact on cropland^46^. Such a strategy lends itself to multiscale data assimilation (for example, highly resolved weather data, or subwatershed HUC12 data, both of which are publicly available). Our future research will also focus on developing interpretability mechanisms for black-box models such as ours to further gain scientific and economic insights from data^47^.

## Materials and methods

### Problem formulation

As described in the Results section, the variable to be predicted in this work is the yield, which is a yearly data and defined at county level in this work. The input variable for the prediction model include hourly weather data, soil quality data, and soil moisture data. Therefore, the yield prediction is defined as $\mathrm{Y}=\mathrm{f}(\mathrm{X})$, where Y is the vector of the yield in county-level, X is the input matrix with multivariate time-series data.

To best extract the information embedded in the time-series data, Long short-term memory (LSTM) networks is selected as it is efficient in dealing with time-series data and extracting recurrent features in relatively long-term time-series data. With this setup, early prediction can also be formulated using the available time-series data, i.e., the weather data prior to August can be used for early prediction in August.

### Recurrent neural network

LSTM is based on Recurrent Neural Network (RNN) and with a special memory cell to handle the features needed to be remembered and those should be discarded. In general, RNN is a family of neural networks for processing sequential data. It is typically used in text prediction and speech recognition.

For a regular RNN, the network consists of three layers: an input layer, a hidden layer and an output layer. $x^{(t)}$ is the input sequence, $y^{(t)}$ is the output sequence and $h^{(t)}$ is a series of hidden states. The number of the hidden layers is not constrained to one. In the deep learning recurrent neural networks, the number of the hidden layers can reach eight or more. Adding hidden layers can help to study the more complex structure of the model, but also requires more data. $U,V,W$ are shared weights that we need to learn. And there is an activation function $f$ that $h^{(t)} = f(Ux^{(t)} + Wh^{(t - 1)} )$, which should be chosen before learning the networks. The corn yield prediction problem could not fit into a regular RNN, therefore we use the many-to-one RNN model here. The many-to-one RNN model is suitable when there is sequence input with one output, thus it is perfectly match with our data format described in the data section.

### Long short-term memory (LSTM)

The mathematical challenge of learning long-term dependencies in recurrent networks is called the “vanishing gradient problem”. As we propagate the gradient back in time, the magnitude quickly decreases. That is to say, as the input sequence gets longer, it becomes more difficult to capture the influence from the first stage. The gradients to the first several input points vanish and are approximately equal to zero (rarely the gradients will explode with much damage to the optimization). Therefore, a special RNN model called Long Short-Term Memory (LSTM) was developed. LSTM uses the identity function with a derivative of one. As a result, the back propagated gradient remains constant instead of vanishing or exploding when passing through. Clearly LSTM has a more complex structure to capture the recursive relationship between the input and hidden layer^48^. We call the cell between the input and hidden layer LSTM cell.

LSTM adds a new sequence $\{ c_{t} \}$ called cell state to RNN. Cell state is a space specifically designed for storing past information (i.e., the memory space) that mimics the way the human brain manipulates information when making decisions. The left part of the cell is the forget gate layer, which makes the decision whether past information stored in the cell state should be discarded or not. The middle is the input gate layer, which decides whether new information from the input should be added or not. The operation is executed to update old cell state $c_{t - 1}$ to $c_{t}$. This is when old information is dropped and new information added. We can get the output as $h_{t}$ by operating the right part, which is the same process as regular RNN.

In summary, the behavior of the memory cell is determined by three gates: input $i_{t}$, output $o_{t}$, and forget $f_{t}$. The updated equations are as follows:

$${i}_{t}=sigmoid({W}_{i}{h}_{t-1}+{U}_{i}{x}_{t}+{b}_{i})$$

$${f}_{t}=sigmoid({W}_{f}{h}_{t-1}+{U}_{f}{x}_{t}+{b}_{f})$$

$${o}_{t}=sigmoid({W}_{o}{h}_{t-1}+{U}_{o}{x}_{t}+{b}_{o})$$

$${\stackrel{\sim }{c}}_{t}=\mathit{tan}h({W}_{c}{h}_{t-1}+{U}_{c}{x}_{t}+{b}_{c})$$

$${c}_{t}={f}_{t}\odot {c}_{t-1}+{i}_{t}\odot {\stackrel{\sim }{c}}_{t}$$

$${h}_{t}={o}_{t}\odot \mathit{tan}h({c}_{t})$$

where all $U\in {\mathbb{R}}^{d\times d},W\in {\mathbb{R}}^{d\times k},b\in {\mathbb{R}}^{d}$ are learnable parameters and the operator $\odot$ denotes the element-wise multiplication.

Figure 2 shows the structure of our final LSTM model used for county-level corn yield prediction in the Corn-Belt.

### Training of LSTM

Loss function is a measure of how good a prediction model does in terms of being able to predict the expected outcome. The loss function we picked for our LSTM model is the mean squared error (MSE). The target of training the prediction model is to find parameters that could achieve the minimum point of the loss function, thus turning it into an optimization problem. The algorithm to learn the recurrent neural network is gradient descent^49^ and back-propagation through time^50^ (BPTT). Gradient descent is one of the most popular algorithms to perform optimization. It is an efficient algorithm to search for the local minimum of the loss function. The BPTT algorithm is used to compute the gradient for the equation $h^{(t)} = f(Ux^{(t)} + Wh^{(t - 1)} )$ and the loss function. The core idea behind BPTT is the composite function chain rule. The nodes of our computational graph include the parameters $U,V,W$ and constant terms as well as the sequence of nodes indexed by $t$ for $x^{(t)}$ and $h^{(t)}$. Once the gradients on the internal nodes of the computational graph are obtained, we can obtain the gradients on the parameter nodes. The parameters are shared across time steps. Given a starting point, calculating the gradient of that point and searching in the direction of the negative gradient is the fastest way to search for a local minimum. Then we can update the parameters with iterations of the gradient descent optimizer by searching for a smaller local minimum.

### Model settings

There are still some questions about the model settings. Should we use one, two, or more hidden layers? Will more related input variables improve the prediction? Will more created training samples generated with combinations described in the data section improve the prediction? We tried all these different settings in the Iowa model and the results showed that the best model is the one with two hidden layers, ten variables picked up in the data augmentation section, and two county combination samples added, which totals 19,734 samples.

How should we expand the model to other states in the Corn-Belt from Iowa? We have three choices: (*a*) apply the “best” Iowa model to all other states; (*b*) train models separately for each state; and, (*c*) train the “best” model with all data from the Corn-Belt. The Iowa model performed badly when applied to the other states. The Corn-Belt model also performed poorly. Even though we include all data from different states, many unknown variables correlated with each state did not get included in the model. LSTM then only keeps the common information and throws away all other noises, leading to the result that our big Corn-Belt model learns less information than does a separate model for each state. Therefore, the best choice and the one we pursued is to train models separately for each state.

### Implementation

Our LSTM model was learned using the Keras^51^ package. We assigned a linear relation between the hidden and output layers. There are several choices for gradient descent optimizer in Keras. We tried both Stochastic Gradient Descent (SGD) and RMSprop optimizer. Our final LSTM model used RMSprop optimizer as this optimizer is usually a good choice for RNNs. Besides the parameters that we need to learn from the data, there is also another kind of parameter specified manually for LSTM models, called a hyperparameter. A model hyperparameter is a configuration that is external to the model whose value cannot be estimated from data. Hyperparameter searching is an important process before the commencement of the learning process. The choice of the hyperparameter influences the learning result.

The hyperparameters that we decided manually for our LSTM model include the number of hidden nodes within each hidden layer, batch size, dropout rate, learning rate, momentum, and decay rate. Batch size is the number of training examples utilized in one iteration of SGD or RMSprop optimizer. The higher the batch size, the more memory space needed. Dropout is a technique where randomly selected neurons are ignored during training. A 0.2 dropout rate means that one in five hidden neurons will be randomly excluded from each updated cycle. Dropout could make the network less sensitive to the specific weights of neurons, and in turn solve the overfitting problem. Learning rate, momentum, and decay rate are important parameters for SGD optimizer. They would decide the speed of convergence of the network. The learning rate is how quickly a network abandons old beliefs for new ones. With a large learning rate, we take huge jumps to reach the bottom. There is also a possibility that we will overshoot the global minima (bottom) and end up on the other side of the pit instead of the bottom. Thus, we will never be able to converge to the global minima. However, it will take too much time to converge if the learning rate is too small. Hence, it is often useful to reduce the learning rate as the training progresses, which is what the decay rate is used for. Momentum is an argument in SGD optimizer to obtain faster convergence. RMSprop optimizer is similar to the SGD optimizer with momentum. It uses a moving average of squared gradients to normalize the gradient itself. We only need to define the learning rate for RMSprop optimizer.

It is impossible to know the best value for a model hyperparameter of a given problem. We may use rules of thumb, copy values used on other problems, or search for the best value by trial and error. What we did was assign a set of numbers by experience for these hyperparameters and let the machine randomly pick one value in the set for each hyperparameter. The choice sets include: (1) hidden nodes, [8, 16, 32, 64, 128, 214], (2) batch size, [16, 64, 128, 512, 1024], (3) dropout rate, [0.0, 0.1, 0.2, 0.3, 0.4, 0.5, 0.6, 0.7], (4) learning rate, [1e−07, 1e−06, 1e−05, 1e−04, 0.001], (5) momentum, [0.0001, 0.001, 0.01,0.05, 0.1], and (6) decay, [0.0001, 0.001, 0.01,0.05, 0.1]. For each choice set, SGD and RMSprop optimizers are tested. Usually after searching for over 300 models with different combinations of hyperparameter settings, we can find the ‘best’ model and the corresponding ‘best’ hyperparameters.

## References

[1] https://apps.fas.usda.gov/psdonline/app/index.html#/app/home

[2] Akerlof GA (1970). The market for “lemons”: quality uncertainty and the market mechanism. Q. J. Econ..

[3] https://medium.com/descarteslabs-team/advancing-the-science-of-corn-forecasting-350603e3c57f.

[4] https://www.cargill.com/story/how-descartes-and-agribusiness-giant-are-unlocking-data-future.

[5] https://www.agweb.com/article/corn-could-be-on-track-for-1662-bu-NAA-ben-potter.

[6] Adjemian, M., Arita, S., Breneman, V., Hungerford, A. & Johansson, R. Market reaction to USDA’s august corn crop reports. farmdoc daily (9):186, Department of Agricultural and Consumer Economics, University of Illinois at Urbana-Champaign, October 4, 2019.

[7] Thomas Reuters, Lanworth Crop Production Forecasts. https://www.thomsonreuters.com/en.html.

[8] Tellus Labs, Kernel. https://telluslabs.com/pages/kernel.

[9] Climate Corp. https://www.climate.com/.

[10] Corn-Belt. Encyclopedia Britannica Online.

[11] https://www.cmegroup.com/trading/agricultural/grain-and-oilseed/soybean.html .

[12] https://www.nass.usda.gov/Publications/Todays_Reports/reports/cropan19.pdf.

[13] Basso B, Liu L (2019). Seasonal crop yield forecast: methods, applications, and accuracies. Adv. Agron..

[14] Basso B, Ritchie JT, Pierce FJ, Braga RP, Jones JW (2001). Spatial validation of crop models for precision agriculture. Agric. Syst..

[15] Charles LH, Benjamin Cook J (2014). “Predicting Yield in the Corn Belt.” Stat 225.

[16] Gerlt S, Thompson W, Miller DJ (2014). Exploiting the relationship between farm-level yields and county-level yields for applied analysis. J. Agric. Resour. Econ..

[17] Storm H, Baylis K, Heckelei T (2020). Machine learning in agricultural and applied economics. Eur. Rev. Agric. Econ..

[18] Shekhar, S., Schnable, P., LeBauer, D., Baylis, K., & VanderWaal, K. Agriculture big data (AgBD) challenges and opportunities from farm to table: a midwest big data hub community whitepaper. NSF Midwest Big Data Hub (2017).

[19] Hochreiter S, Schmidhuber J (1997). Long short-term memory. Neural Comput..

[20] Goodfellow I, Bengio Y, Courville A (2016). Deep Learning.

[21] Michael H, Peter S (2003). Recurrent neural networks for time series classification. Neurocomputing.

[22] Che, Z., Sanjay, P., Cho, K., Sontag, D. & Liu, Y. Recurrent Neural Networks for Multivariate Time Series with Missing Values. ICLR (2017). 10.1038/s41598-018-24271-9 PMC5904216 29666385

[23] Fred, B. The Seven Wonders of the Corn Yield World. Institute of Ag Professionals (2008).

[24] Foote RJ, Bean LH (1951). Are yearly variations in crop yields really random?. J. Agric. Econ. Resour..

[25] Kaylen MS, Koroma SS (1991). Trend, weather variables, and the distribution of U.S. corn yields. Rev. Agric. Econ..

[26] Deschênes O, Greenstone M (2007). The economic impacts of climate change: evidence from agricultural output and random fluctuations in weather. Am. Econ. Rev..

[27] Schlenker W, Roberts MJ (2009). Nonlinear temperature effects indicate severe damages to U.S. crop yields under climate change. Proc. Natl. Acad. Sci..

[28] Yu, T. *Three Essays on Weather and Crop Yield*. Ph.D. thesis, Economics, Iowa State University (2011).

[29] Lobell, D. B., et al. Eyes in the sky, boots on the ground: assessing satellite-and ground-based approaches to crop yield measurement and analysis. Am. J. Agric. Econ. (2019).

[30] Monisha K, Robert L, Charles W (2005). Artificial neural networks for corn and soybean yield prediction. Agric. Syst..

[31] Newlands, N. K. & Townley-Smith, L. Predicting energy crop yield using Bayesian networks. In *Proceedings of the Fifth IASTED International Conference, Computational Intelligence* 107–112 (2010).

[32] Chawla, V., Naik, H.S., Akintayo, A., Hayes, D., Schnable, P., Ganapathysubramanian, B., and Sarkar, S. A Bayesian Network Approach to County-Level Corn Yield Prediction using Historical Data and Expert Knowledge. In *KDD 16 Workshop: Data Science for Food, Energy and Water, San Francisco* (ISBN: 978-1-4503-2138-9) (2016).

[33] Kim NL, Lee Y-W (2016). Machine learning approaches to corn yield estimation using satellite images and climate data: a case of Iowa State. J. Korean Soc. Surv. Geod. Photogramm. Cartogr..

[34] USDA., NASS Quickstats. https://quickstats.nass.usda.gov/.

[35] Li, Lisha, *Three Essays on Crop Yield, Crop Insurance and Climate Change* (2015).Graduate Theses and Dissertations. 14364.https://lib.dr.iastate.edu/etd/14364.

[36] Li, L. *The Impact of Weather and Weather Extremes on Corn Yields*. Ph.D. thesis, Economics, Iowa State University (2015).

[37] Weather Underground. https://www.wunderground.com/history/.

[38] NDAWN Center. https://ndawn.ndsu.nodak.edu/help-corn-growing-degree-days.html/.

[39] Historical Palmer Drought Indices, NOAA. https://www.ncdc.noaa.gov/temp-and-precip/drought/historical-palmers/.

[40] AgriData, NRCS Soil Map Data. https://www.agridatainc.com/Home/Products/Surety/NRCS%20Online%20Soil%20Maps.

[41] USDA., SSURGO. https://www.nrcs.usda.gov/wps/portal/nrcs/detail/soils/survey/geo/?cid=nrcs142p2_053631.

[42] U.S. Geological Survey, Department of the Interior. https://catalog.data.gov/dataset/national-land-cover-database-nlcd-land-cover-collection.

[43] Hendricks NP (2018). Potential benefits from innovations to reduce heat and water stress in agriculture. J. Assoc. Environ. Resour. Econ..

[44] Juan JA, Fazli M, Zhou XM (2008). Climate change, weather variability and corn yield at a higher latitude locale. Southwest. Quebec. Clim. Change.

[45] Peng H, Long F, Ding C (2005). Feature selection based on mutual information criteria of max-dependency, max-relevance, and min-redundancy. IEEE Trans. Pattern Anal. Mach. Intell..

[46] Zhang Y, Chipanshi A, Daneshfar B, Koiter L, Champagne C, Davidson A, Reichert G, Bédard F (2019). Effect of using crop specific masks on earth observation based crop yield forecasting across Canada. Remote Sens. Appl. Soc. Environ..

[47] Zilberman D (2019). agricultural economics as a poster child of applied economics: big data & big issues. Am. J. Agr. Econ..

[48] Lei J, Liu C, Jiang D (2019). Fault diagnosis of wind turbine based on Long Short-term memory networks. Renew. Energy.

[49] Léon B (1998). Online Algorithms and Stochastic Approximations. Online Learning and Neural Networks.

[50] Mozer MC (1995). A Focused Backpropagation Algorithm for Temporal Pattern Recognition. Backpropagation: Theory, architectures, and applications. ResearchGate.

[51] Keras Documentation. https://keras.io/.

